# Methodological challenges in Dutch HTA of non-oncological orphan drugs: a retrospective analysis and price comparison using different pricing models

**DOI:** 10.1186/s13023-025-04181-6

**Published:** 2026-01-08

**Authors:** Jelle Walraven, Mahtab Kaveh, Carin Uyl - de Groot

**Affiliations:** 1 Erasmus School of Health Policy & Management, Burgemeester Oudlaan 50, Rotterdam, 3062 PA Netherlands; 2 Sanofi, Paasheuvelweg 25, Amsterdam, 1105 BP Netherlands

**Keywords:** Health economic assessment, Cost-effectiveness analysis, Pharmaceutical pricing, Orphan drugs, Orphan diseases

## Abstract

**Background:**

Cost-effectiveness analyses can have limited informative value for pricing and reimbursement decisions for orphan drugs. In cases where cost-effectiveness cannot be reliably assessed or achieved, value-based pricing principles may not be applicable. As a result, alternative pricing models have been proposed. It remains unclear how these alternative approaches compare to one another and to traditional value-based pricing. This study aims to explore and compare these pricing models in the context of orphan drugs.

**Methods:**

All cost-effectiveness assessments of non-oncological orphan drugs published by the Dutch National Health Care Institute between 2015 and 2024 were analyzed to identify methodological challenges and recommended value-based price estimates. For each treatment, prices were also estimated using a cost-plus pricing model and a discounted cash flow model. These estimates were then compared to value-based prices and public list prices.

**Results:**

Cost-effectiveness assessments of 13 different therapies were found, 12 of which provide information for determining a value-based price. All assessment reports cite major uncertainties or unresolved issues in one or more of the following areas: (1) lack of a suitable comparator, (2) sub-optimal disease understanding, (3) limited evidence to inform models, (4) effect uncertainty and (5) flawed QoL measurement. Only one single treatment was found to be cost-effective at the appropriate threshold. Value-based prices were found to fall below, within or above the price ranges of the two alternative models.

**Conclusion:**

Challenges cited in literature are present in Dutch assessments of the cost-effectiveness of orphan drugs. Although these issues cause considerable uncertainty, they did not negate CEA’s ability to inform decision-making. Still, orphan drugs tend to be far from cost-effective, providing a challenge for patient access that is both timely and financially feasible. Alternative models like cost-plus pricing and discounted cash flow tend to generate even lower price estimates and rely on considerable assumptions, making them unlikely to offer a viable solution in their current state.

**Supplementary information:**

The online version contains supplementary material available at 10.1186/s13023-025-04181-6.

## Introduction

Cost-effectiveness analysis (CEA) is a prevalent method to help inform decision-makers regarding pricing and reimbursement of new therapies. For new pharmaceutical therapies seeking reimbursement on an added value claim in the Netherlands, CEA is mandatory [1]. The central outcome of interest is the incremental cost-effectiveness ratio (ICER), which represents the ratio between incremental costs and incremental health benefits, typically expressed as the additional cost per QALY gained. The ICER is benchmarked against a predefined willingness-to-pay threshold to determine the maximum price that ought to be paid for a treatment and what discount is required on the initial list price to justify reimbursement. This approach is known as value-based pricing. Although CEA is not the only factor in reimbursement decisions, it plays a significant role, as the ICER is used to inform price negotiations between manufacturers and payers.

When it comes to orphan drugs, however, two issues undermine the informative value of CEA for pricing and reimbursement decisions. First, the inherent characteristics of rare diseases complicate health economic assessments, reducing the feasibility of robust economic evaluations and increasing uncertainty [2]. Second, orphan drugs typically do not show cost-effectiveness under the traditional economic evaluation criteria [2–4]. HTA agencies understand, and sometimes even accept, these limitations of value-based pricing for orphan drugs. Still, when cost-effectiveness cannot be adequately assessed or achieved, value-based pricing can pose an obstacle to timely access, CEA’s ability to reliably estimate a price that should be acceptable to both payers and pharmaceutical companies is hindered.

As a result, other approaches, such as cost-plus pricing (CPP) and the discounted cash flow (DCF) method have recently been suggested in case conducting a CEA is unfeasible or when a drug fails to realistically meet the cost-effectiveness threshold [5, 6]. Interestingly, these models for determining ‘fair’ or ‘reasonable’ prices for (orphan) drugs have been published from opposing perspectives, despite using similar parameters. One approach seeks to determine the maximum price the payer should be willing to pay, whereas the other estimates the minimum price that pharmaceutical companies should consider acceptable. These new pricing models have seen little application and minimal comparison with the more value-based pricing approach.

This paper describes the challenges incurred with CEA-informed value-based pricing of orphan drugs in literature and Dutch HTA practice. Additionally, it presents price estimates using two alternative approaches which are compared to value-based prices in the Netherlands.

## Background

Although the methodological challenges and low probability of cost-effectiveness associated with orphan drugs are not new, they are becoming increasingly relevant to pricing and reimbursement conditions, as illustrated by two recent cases in the Netherlands: atidarsagene autotemcel and olipudase alfa.

### Methodological challenges

A number of different factors inherent to orphan drugs can negatively impact the practical feasibility and uncertainty of CEA [7]. A practical example from the Netherlands is the case of atidarsagene autotemcel. Limited data availability caused considerable uncertainty about utilities and treatment effectiveness between patient subgroups. Disagreement on response classification and treatment effect durability further complicated the assessment [8]. Despite the payer’s willingness to accept a higher price and thus exceeding the willingness-to-pay threshold due to the unique circumstances, no agreement on an acceptable price could initially be reached. Reimbursement of atidarsagene autotemcel was temporarily declined as a result.

### Unfavourable cost-effectiveness

Although proper pharmacoeconomic modelling practices may address these inherent issues to a certain extent, most (ultra-)orphan drugs will not meet conventional criteria for cost-effectiveness [3]. While there seems to be a certain willingness to get these therapies to patients despite their uncertainty, high cost and low probability of being cost-effective both in general [4] and in the Netherlands specifically [9], there seems to be a limit to such tolerance.

A recent example is olipudase alfa. The CEA report submitted by the Marketing Authorization Holder (MAH) was not formally assessed by the HTA agency the Dutch National Healthcare Institute (ZIN), as the submitted ICER’s of €1.070.000 and €1.600.000 clearly indicated the treatment is not cost-effective. No conclusions were drawn regarding the quality of the analysis, but a full assessment of the treatment’s cost-effectiveness was not expected to result in a reliable price estimate. Olipudase alfa was nonetheless recommended for reimbursement on the condition that price negotiations would result in a substantial discount in order to approach a more cost-effective price [10].

### Alternative pricing approaches

In cases where cost-effectiveness cannot be properly assessed or reasonably achieved, and value-based pricing principles can thus not be applied, a non-arbitrary method is still needed to inform prices that are justifiable for both the MAH as well as the payer, given that market principles do not apply in this context. Two alternative approaches have recently been proposed: cost-plus pricing and the discounted cash flow method.

#### Cost-plus pricing

Certain researchers have argued that if an orphan drug does not meet the cost-effectiveness threshold, other mechanisms should be used to determine a just and reasonable price [5]. This approach considers the interest of payers as it seeks to determine a maximum price that ought to be paid to acquire these therapies. Since pharmaceutical firms cite higher average costs in justification of higher orphan drug prices, the price is based on the incurred costs and a pre-determined profit margin. Certain authors have applied CPP models to specific orphan drugs [11, 12]. The International Association of Mutual Benefit Societies has proposed a CPP model with broad applicability, encompassing orphan drugs as well as other therapies and therapeutic areas [13].

#### Discounted cash flow

Other authors have adopted a supply-side perspective, postulating that pharmaceutical innovation is driven by profit-seeking companies and that drug prices must be sufficiently high for companies to commit to such a risky endeavour [14]. They argue that the business valuation principles guiding investment decisions in the pharmaceutical industry could also be utilized for public price setting. When the ICER exceeds the threshold, a DCF analysis could be performed in which expected future returns are contrasted to upfront investment using a risk-adjusted capital cost threshold. This could help determine a price at which companies are sufficiently incentivized to develop new drugs for rare diseases [15].

#### Comparing both approaches

Strikingly, both CPP and DCF rely on similar determinants, despite the fundamentally opposing views on what forms the basis of a reasonable price. It is, however, unclear how the price estimates resulting from these two approaches compare to each other and to the traditional value-based pricing method in the context of orphan drugs. This paper aims to address just that matter.

## Methods

All CEA reports on non-oncological orphan drugs published by ZIN between 2015 (introduction of the new ‘lock’ HTA procedure) and 2024 were collected in order to identify methodological challenges. Prices for these treatments were then re-determined using the CPP model by AIM [13] and the DCF model by Nuijten et al. [14, 15] and compared to the value-based prices and public list prices.

### Analysis of CEA reports

A complete list of Dutch HTA assessments was extracted from the pharmaceutical lead time dashboard of the Dutch Ministry of Health [16] and cross-referenced with EMA’s register of orphan designations [17]. Matches were manually checked for pharmacoeconomic reports. All resulting reports were systematically reviewed by both authors for explicitly mentioned methodological challenges specific to orphan drugs. These challenges were then categorized as (1) lack of a suitable comparator (2), sub-optimal disease understanding (3), limited evidence to inform models (4), effect uncertainty or (5) flawed QoL measurement.

### CPP and DCF price estimates

For the price estimates, Excel versions of the CPP model by AIM and the DCF model by Nuijten et al. were obtained through their respective developers. Both models share the same key variables, which are outlined in Table 1. The approach differs in two aspects, however. The first lies in the conceptualization of expected return. AIM’s CPP model allows for a fixed profit margin whereas the DCF model employs a risk-adjusted discount rate. In both models, the same base percentage is used. The second is probability of success, which is only included in the DCF model. The DCF model explicitly incorporates the probabilities of successfully progressing through the stages of clinical development, whereas the CPP approach assumes that the cost of failure is already embedded in the overall R&D investment. We note that incorporating previous failure costs assumes that a company has the resources and capabilities to develop alternative assets to mitigate potential losses, which may not always hold true for firms developing orphan drugs. To facilitate comparison between both models, equal values are used. Since no data is available on the R&D investment for all therapies, the default minimum and maximum values of AIM’s model (€250.000.000 and €2.500.000.000) were used to establish a range. Of these R&D expenditures, 42% is attributed to Europe, as it represents 42% of the population of main markets for the innovative drugs. Subsequently, 4.58% is attributed to the Netherlands as AIM advocates differential pricing based on gross domestic product per capita [13]. Table 1 provides an overview of the common input parameters and their respective sources. The exact values for the production costs, innovation bonus and patients treated for each treatment are provided in appendix A as they are different for each therapy.

**Table 1:** Model input parameters

Variable	Value	Reference
Research and development investment	€ 250.000.000 and € 2.500.000.000	International Association of Mutual Benefit Societies
Production costs	Varies per therapy (Appendix A)	Treatment summary of product characteristics
Sales and medical information	30%	International Association of Mutual Benefit Societies
Expected return	8%	International Association of Mutual Benefit Societies
Innovation bonus	Varies per therapy (Appendix A)	HTA’s therapeutic assessment report
Allocation base for the Netherlands	1,9236%	International Association of Mutual Benefit Societies
Patients expected to be treated in the Netherlands	Varies per therapy (Appendix A)	HTA’s budget impact report
Duration of therapy	Varies per therapy (Appendix A)	HTA’s budget impact report
Success rate	100%	Assumed since failure costs are already incorporated

### CEA price estimates

Value-based prices for the treatments were estimated by applying the discount required to achieve cost-effectiveness at the relevant threshold to the list price for each treatment. The value-based price of cannabidiol is based on the average dose scenario rather than the maximum dose scenario. For nusinersen, only the SMA type 2/3 subgroup was considered, as the type 1 subgroup analysis did not result in a meaningful recommended discount.

## Results

### Orphan drugs with CEA

**Fig. 1:**
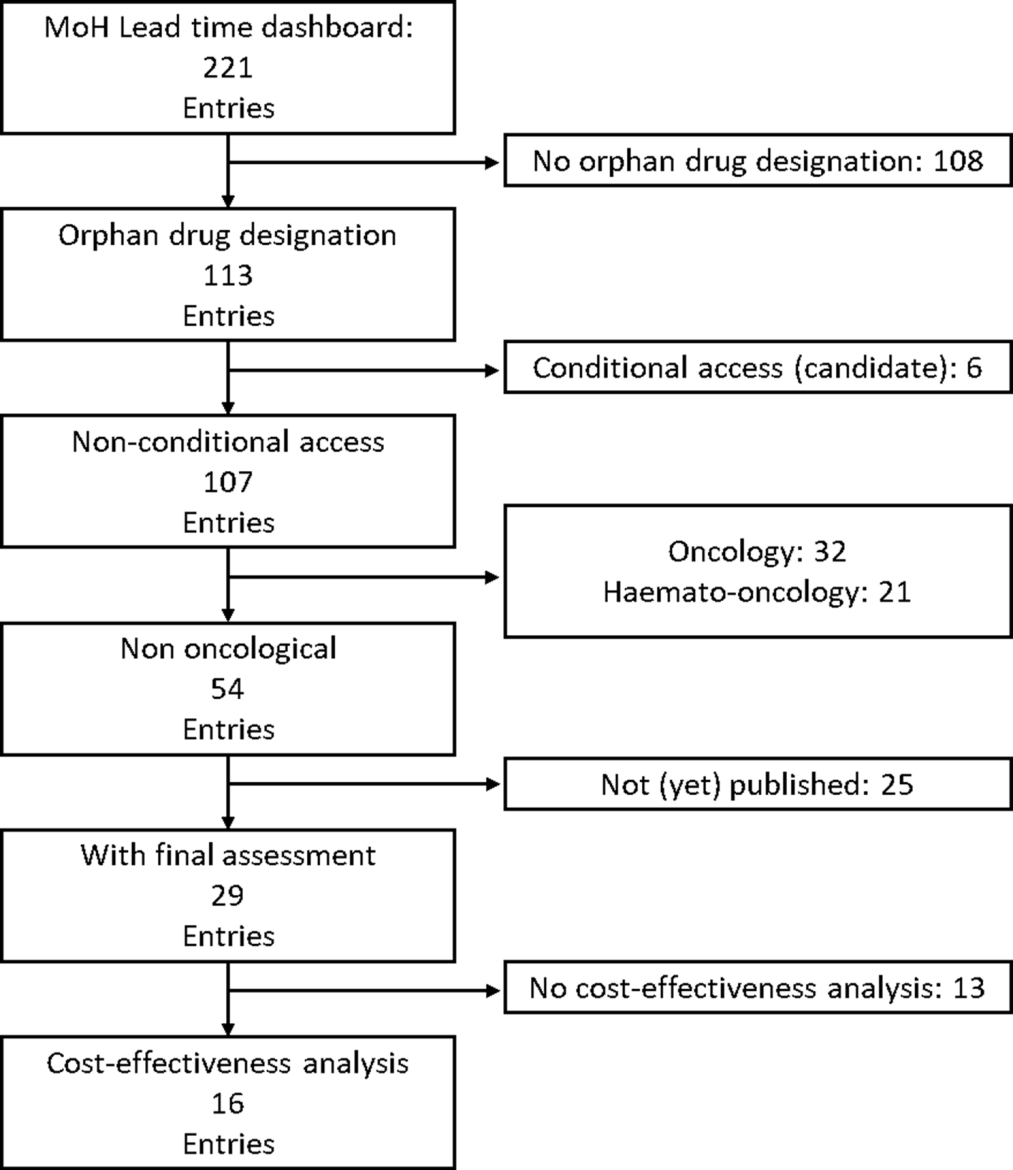
Flow diagram of included reports selection

A complete set of non-oncological orphan drugs with a pharmacoeconomic assessment was obtained through the steps outlined in Fig. 1. Oncological drugs were excluded since they may hold orphan status for certain indications whereas other indications are not granted such a status. Through this process, 16 published HTA reports with a CEA were found on 13 therapies: 13 original submissions and 3 resubmissions as a result of insufficient methodology of the original submission. One report was accepted despite the structural uncertainty making the results unfit for decision making because it was deemed unlikely that resubmission and reassessment would lead to sufficient improvement [18]. Another report was considered suitable for decision-making despite ZIN’s limited confidence in the CEA, as there was high certainty that the therapy’s probability of being cost-effective was 0% [19]. The reports contain the CEA results of 13 different treatments for 19 patient sub-groups, providing ICER’s for 18 and yielding value-based prices for 15. An overview of these results is presented in Table 2.

**Table 2:** Overview of included cost-effectiveness assessment reports

Substance name	Sub-group(s)	HTA report publication	Comment	Reimbursement status in the Netherlands	Status orphan Designation	Treatment cost first year*	Average treatment cost over 10 years*	Reference value	ICER (QALY), deterministic, discounted	ICER (QALY), probabilistic, discounted	Cost effectiveness probability	Discount required to achieve cost-effectiveness
Avacopan	Granulomatosis with polyangiitis and microscopic polyangiitis	15–6-2023	Reassessment	Reimbursed	Active	€70.080	€70.080	€20.000	€365.614	€351.836	6%	80%
		20–4-2022						-	Not meaningful	Not meaningful	Not meaningful	N.a.: insufficient methodological quality
Atidarsagene autotemcel	Presymptomatic late-infantile (1)	27–9-2022	BeNeLuxA joint assessment	Reimbursed	Active	€2.875.000	€287.500	€80.000	€462.632	Not provided	Not provided	85%
	Presymptomatic early juvenile (2)			Reimbursed	Active			€80.000	€225.400	Not provided	Not provided	60%
	Early symptomatic early juvenile (3)			Not reimbursed	Active			€80.000	€396.882	Not provided	Not provided	N.a.: negative advice
Pegcetacoplan	Paroxysmal Nocturnal Hemoglobinuria	16–9-2022		Reimbursed	Active	€316.050	€316.050	€20.000	€1.668.496	Not meaningful	Not meaningful	85% ***
Risdiplam	Spinal muscular atrophy type 1 (1)	15–7-2022		Reimbursed	Withdrawn	€256.835 **	€256.835 **	€80.000	€362.300	Not provided	Not provided	94%
	Spinal muscular atrophy type 2/3 (2)			Partially reimbursed	Withdrawn			€80.000	€416.471	Not provided	Not provided	78%
Cannabidiol	Dravet syndrome	4–7-2022		Reimbursed	Active	€29.935	€29.935	€80.000	€37.584	€38.740	77%	20% ****
	Lennox-Gastaut syndrome			Reimbursed	Active			€80.000	€70.561	€71.124	59%	
Tafamidis	Wild type or hereditary transthyretin amyloidosis	11–8-2021	Reassessment	Reimbursed	Active	€122.552	€122.552	€80.000	€151.050	€153.847	2%	50%
		3–12-2020		-	-			-	Not meaningful	Not meaningful	Not meaningful	N.a.: insufficient methodological quality
Onasemnogene abeparvovec	Spinal muscular atrophy type 1	6–5-2021	BeNeLuxA joint assessment	Reimbursed	Active	€1.945.000	€194.500	€80.000	€263.389	Not meaningful	Not meaningful	91% ***
Ivacaftor/ tezacaftor/ elexacaftor	Cystic fibrosis in patients with homozygous delta F508 mutation (1)	29–4-2021		Partially reimbursed	Active	€220.500	€220.500	€50.000	€210.695	€227.377	0%	75%
	Cystic fibrosis in patients with heterozygous delta F508 mutation (2)			Partially reimbursed	Active			€80.000	€283.991	€297.800	0%	70%
Givosiran	Acute hepatic porphyria	2–2-2021		Reimbursed	Active	€552.322	€552.322	€50.000	€199.836	€196.170	0%	40%
Tezacaftor/ivacaftor	Cystic fibrosis in patients with heterozygous delta F508 mutation	11–08-2020		Reimbursed	Active	€185.127	€185.127	€80.000	€376.060	€378.010	0%	80%80%
Voretigene neparvovec	Inherited retinal dystrophy caused by confirmed biallelic RPE65 mutations	17–2-2020		Reimbursed	Active	€ 690.000	€69.000	€50.000	Not meaningful	Not meaningful	Not meaningful	N.a.: insufficient methodological quality
Nusinersen	Spinal muscular atrophy type 1 (1)	7–2-2018		Conditionally reimbursed	Active	€249.900	€249.900	€80.000	€502.289	€503.740	0%	N.a.: at 100% discount still not cost-effective
	Spinal muscular atrophy type 2/3 (2)			Conditionally reimbursed	Active			€80.000	€1.059.269	€1.082.249	0%	85%
Lumacaftor/ivacaftor	Cystic fibrosis in patients homozygous delta F508 mutation	15–12-2016	Belgium & Netherlands joint assessment; reassessment	Reimbursed	Withdrawn	€169.386	€169.386	€80.000	€402.883	€387.541	0%	82%
		13–5-2016		-	-			-	Not meaningful	Not meaningful	Not meaningful	N.a.: insufficient methodological quality

* Based on list price at date of submission, 100% adherence, maintenance dose

** For patients above the age of 2 that weigh more than 20 kilograms

*** Referenced to comparator price after applying discount required for the comparator to be cost-effective

**** Discount based on an alternative CEA scenario deemed more realistic by the HTA agency

### Methodological challenges

ICER’s estimated for 12 out of the 13 treatments were deemed appropriate for reimbursement decision-making, despite the methodological challenges and uncertainty involved. All reports mentioned major sources of uncertainty or unresolved discussion points listed in the concluding section of the assessment report, which addresses transparency, methodology, inaccuracy, bias, and lack of evidence. One of the CEA models was described as having a subjective character as a result of the heavy reliance on judgement-based estimates for both utilities and costs [20]. Identified challenges were grouped into five overarching categories.

#### Lack of a suitable comparator

Only three treatments [21–23] were compared head-to-head to another treatment for at least one patient subgroup. One other treatment was compared with the standard of care which consisted of a variety of pharmaceutical and non-pharmaceutical care [20]. The remaining treatments were compared with best supportive care (while also being added to best supportive care). In one instance [24], an attempt was made to compare trial results with a competing treatment. However, the small study populations introduced bias and significant uncertainty, making the data unsuitable for reliable comparison. Three therapies were assessed using only data from single-arm studies [8, 24, 25] for at least one patient sub-group, as a consequence of the extreme rarity of the disease or ethical considerations involving young infants. CEA’s, by definition, seek to compare two treatment alternatives. The absence of such an alternative deteriorates the basis for determining the true value of these treatments in comparison to existing alternatives.

#### Sub-optimal disease understanding

A lack of evidence and understanding of the disease pathophysiology and mechanisms lead to systematic uncertainty in the fundamental elements of the model. One frequently mentioned issue concerns the basic definition of health states, resulting in arbitrary definitions of health states [20, 26] or overly complex measures with limited clinical relevance [27]. Another issue reported is the absence of validated measurement instruments for certain patient (age) groups [8, 26] and the inability of existing instruments to capture the full range of outcomes [25]. In two instances, the MAH indicated that the primary measurement instruments were either not sensitive enough to detect clinically relevant changes [25] or not suitable for linking utilities to costs [18].

#### Limited evidence to inform models

Another issue mentioned is the scarcity of gathered data regarding the new treatment or its comparator to populate the model. In one instance, this lack of sufficient data compelled the MAH to a less appropriate modelling method [27]. In another case, the informing study lacked sufficient statistical power to support the model’s health state structure [28]. Lack of evidence on natural disease progression was cited to also complicate the modelling of the control arm and long-term effects [24, 25, 27]. Small populations have also been reported to complicate the extrapolation of survival [22, 24, 29], thus adding more uncertainty to the model outcomes. Other issues cited include medically plausible transitions between health states not being observed in the collected data [18], utility values for health states being deemed unreliable [24, 27] and low confidence in the classification of outcomes [8, 30].

#### Effect uncertainty

The relatively short period of time over which evidence was gathered added to the uncertainty of the assessments. Extrapolation of short-period data carried the risk of overestimation [24, 25] and left unresolved uncertainty regarding the sustainability of the treatment effect [8, 18, 19, 23, 24]. The absence of long-term evidence is sometimes crudely addressed by freezing patients in their last recorded health state, leading to unrealistic assumptions about disease progression [20, 27]. The absence of data on long-term complications is another cited source of uncertainty [22, 27]. This issue is particularly relevant for treatments targeting younger patients, where upfront costs come with expected benefits later in life, leading to considerable uncertainty from a CEA perspective. These challenges introduce considerable uncertainty to both treatment outcomes and costs over a life-long time horizon.

#### Flawed QoL measurement

In Dutch pharmacoeconomic assessments, results are required to be reported in a cost-per-incremental-QALY format, which presents another hurdle. First and foremost, no QoL data were collected in six pivotal clinical studies [8, 18, 20, 24, 25, 30]. In some instances missing QoL data can be justified through the patients’ very young age [8, 24, 25, 30] or inability to communicate effectively due to their condition [20]. Two reports stated that QoL measurements derived from the clinical study were not used because they were argued to be too high as a result of coping [19, 25].

In such cases, alternative approaches like vignette studies [8, 18, 20] were used, which lack methodological robustness and objectivity. In other instances, QoL is not assessed via the preferred EQ-5D questionnaire but through disease specific instruments instead [22, 23, 30]. The results are then ‘mapped’ to EQ-5D derived utilities. The MAH holders have signalled that these approaches may still not be appropriate because of limited sensitivity [20] or poor predictive capabilities [23]. Another cited approach is mapping utility values to health states using utilities derived from a similarly indicated treatment [25] or by employing utility values from other patient sub-groups [24, 30] as proxies. These approaches to QoL measurement have a tendency to produce uninformative utilities with poor face validity. The results can be a clinically implausible amount of worse than death health states [8, 24] or poor differentiation between health states [25].

### Unfavourable cost-effectiveness

Table 2 contains the results from the CEA across all 13 HTA reports. Only one treatment was deemed cost-effective at the relevant threshold. However, the unrealistic and prolonged extrapolation of the treatment effect, the limitations in the data used, and the subjective nature of the model led ZIN to base their recommendation on an alternative scenario that estimated that a 20% discount would be required in order for the treatment to be cost-effective [20]. Another treatment was initially found to dominate the comparator, but the comparator itself was not cost-effective. After applying a recommended discount of 90% to the comparator’s price, the treatment was no longer cost-effective [22]. In reports where such an estimate is provided, the probability of cost-effectiveness is close to zero percent. Such results often lead to disconcerting discount recommendations. For instance, a 100% discount would still not result in the treatment being cost effective at the highest threshold due to the high costs of complementary standard care combined with the survival benefits of the treatment [30].

### Alternative price calculations

**Fig. 2:**
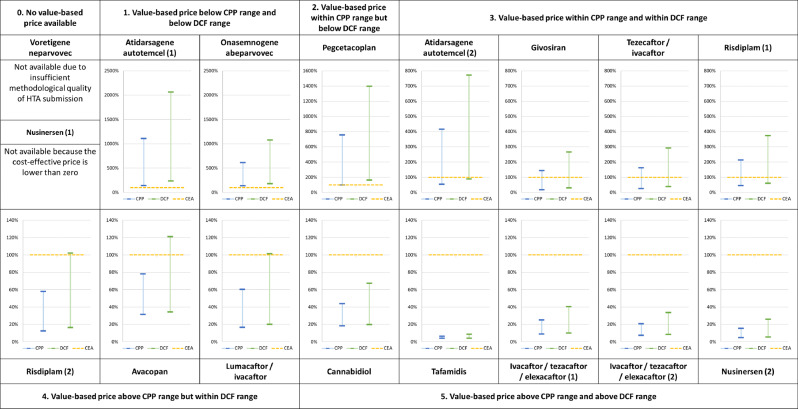
Price estimates for CPP, DCF and CEA

The estimated CPP price, DCF price, value-based price and list price of each treatment are presented in Table 3. Figure 2 visualises these results, expressing the price estimates as a percentage of ZIN value-based price. List prices are excluded from the figure because their values would distort the interpretability of the figure. The percentage values can be found in Appendix B.

**Table 3:** Price estimates for CPP, DCF, CEA and list price

	CPP price		DCF price		CEA price	List price
Substance name	Min	Max	Min	Max	ZIN	MAH
Avacopan	€ 4.414	€ 10.620	€ 4.802	€ 16.973	€ 14.016	€ 70.080
Atidarsagene autotemcel (1)	€ 610.626	€ 4.774.258	€ 1.014.154	€ 8.881.543	€ 431.250	€ 2.875.000
Atidarsagene autotemcel (2)	€ 610.626	€ 4.774.258	€ 1.014.154	€ 8.881.543	€ 1.150.000	€ 2.875.000
Pegcetacoplan	€ 46.615	€ 358.418	€ 76.291	€ 661.662	€ 47.408	€ 316.050
Risdiplam (1)	€ 6.997	€ 32.707	€ 9.276	€ 57.656	€ 15.410	€ 256.835
Risdiplam (2)	€ 6.997	€ 32.707	€ 9.276	€ 57.656	€ 56.504	€ 256.835
Cannabidiol	€ 4.367	€ 10.465	€ 4.715	€ 16.103	€ 23.948	€ 29.935
Tafamidis	€ 3.814	€ 6.278	€ 3.810	€ 8.399	€ 98.042	€ 122.552
Onasemnogene abeparvovec	€ 240.525	€ 1.073.252	€ 314.831	€ 1.888.309	€ 175.050	€ 1.945.000
Ivacaftor/tezacaftor/elexacaftor (1)	€ 4.833	€ 13.768	€ 5.459	€ 22.192	€ 55.125	€ 220.500
Ivacaftor/tezacaftor/elexacaftor (2)	€ 4.833	€ 13.768	€ 5.459	€ 22.192	€ 66.150	€ 220.500
Givosiran	€ 58.163	€ 473.901	€ 97.972	€ 878.467	€ 331.393	€ 552.322
Tezacaftor/ivacaftor	€ 9.171	€ 59.853	€ 13.786	€ 108.160	€ 37.025	€ 185.127
Voretigene neparvovec	€ 344.275	€ 2.290.750	€ 437.104	€ 3.291.040	Not available	€ 690.000
Nusinersen (1)	€ 17.340	€ 57.570	€ 20.579	€ 96.443	Not available	€ 249.900
Nusinersen (2)	€ 17.340	€ 57.570	€ 20.579	€ 96.443	€ 374.850	€ 249.900
Lumacaftor/ivacaftor	€ 5.021	€ 18.353	€ 6.058	€ 30.884	€ 30.489	€ 169.386

The prices estimates derived from the CPP and DCF show significant variation between therapies and between one another. The same holds for the price estimates compared to the value-based prices recommended by ZIN. On first sight, the price estimations resulting from the CPP and DCF models show no consistent relationship to one another or the value-based price. Still, certain relationships can be discerned:

0. Value-based price cannot be determined (2 instances)
1. Value-based price is below CPP range and below DCF range (2 instances)
2. Value-based price is within CPP range but below DCF range (1 instance)
3. Value-based price is within CPP range and within DCF range (4 instances)
4. Value-based price is above CPP range but within DCF range (3 instances)
5. Value-based price is above CPP range and above DCF range (5 instances)

Four findings in particular are of interest. The first of which is defined through category 1, where value-based estimates are below the lowest DCF and CPP estimates. This indicates that, even under the most optimistic circumstances with regard to R&D investment, it would not be feasible to develop and market these therapies at a cost-effective price in the Netherlands. Second, there is a group of therapies where the value-based price far exceeds the CPP and DCF price estimates, even at the highest level of R&D expenditure. Indicating that despite the small target population, development and marketing of the therapy should be feasible at a cost-effective price. Third, the DCF price estimates tend to be higher than the CPP estimates. This does not necessarily have to be the case, however, as is demonstrated by the price estimates for tafamidis when assuming an R&D investment of € 250.000.000. Lastly, some of the DCF and CPP price estimates that assume the maximum R&D investment of € 2.500.000.000 exceed the actual list prices of the treatments as set by the MAHs.

## Discussion

This study examined the methodological issues and reported outcomes of all CEA’s on non-oncological orphan drugs published by ZIN in the period between 2015 and 2024 and compared the value-based price with estimates derived from the CPP and DCF models.

### Analysis of CEA reports

Through a systematic approach, 13 completed HTA reports for non-oncological orphan drugs with a CEA were identified. The reports contain the CEA results of 13 different treatments on 19 patient sub-groups, providing 15 value-based price estimates.

Methodological challenges frequently cited literature are indeed also cited in Dutch HTA reports, although the number of issues and level of detail in which they were discussed differs significantly between assessments. These challenges did not undermine the use of CEA to support decision making, as is supported by an earlier review [4] and the fact that 12 out of 13 CEAs were deemed of sufficient quality to support decision-making. The high level of uncertainty stemming from these challenges may, however, result in an eventual net health loss when the financial risk caused by this uncertainty is borne by the payer.

With the exception of cannabidiol, no treatment was regarded to be cost-effective or to have a probability of cost-effectiveness higher than 6% at their respective thresholds, with the base case estimates for cannabidiol still being open to debate. Consequently, the majority of the recommended discounts exceeded 75%. The high discount recommendations, therapeutic benefits of these treatments and lack of alternatives highlight the paradoxical challenge of obtaining patient access at both timely and at reasonable costs.

### Price estimates

The CPP price estimates are heavily influenced by the ratio of production costs to R&D. This ratio, in turn, is dependent on the compound and the amount of patients. These effects further enhanced depending on the innovation bonus, affecting both the low and high estimates, thereby also determining the range between the two. This explains why tafamidis, a small molecule with high expected patient count and relatively modest innovation bonus, has the smallest price range whereas givosiran has the broadest due to the higher production cost, low patient number and average innovation bonus. Interestingly, under the CPP method treatments with higher production costs are allowed a higher level of profitability in absolute terms than treatments with lower production costs.

As with the CPP model, the DCF price estimates are mostly influenced by expected patient numbers. Increases in R&D expenditure will have a stronger impact on treatments where this increase can be spread out amongst fewer patients. DCF price estimates tend to be higher than those based on the CPP model. This relationship changes, however, as patient numbers increase. This can be attributed to the distinct way each model accounts for profit. In the DCF model, total profit is a fixed proportion of R&D investment. In the CPP model, total profit is the sum of the profit margins per patient and therefore increases as patient numbers rise, all else being equal. This means that sufficiently high patient numbers will cause the CPP price estimate to exceed the DCF estimate. Table 3 illustrates this with tafamidis, an orphan drug with a relatively high expected patient population in the Netherlands.

The value-based prices determined by ZIN show no consistent relationship to the CPP and DCF price ranges. Given the vastly different model determinants, this is unsurprising. Expected patient numbers and production cost are very relevant to CPP and DCF, but of no consequence for CEA. Conversely, factors like willingness-to-pay thresholds and comparator price discounts are not included in the CPP and DCF models. The valuation of clinical effects in CPP and DCF through the use of innovation bonuses is vastly different from the more sophisticated approaches used in value-based pricing to determine incremental clinical value.

### Alternative pricing models

The alternative pricing models, in their current state, appear to have their own limitations and assumptions. First, the data required to perform these price calculations are sparsely available. Pharmaceutical companies are reluctant to be transparent about the real cost and risk involved with developing drugs, which means that industry averages or rules of thumb have to be relied upon, which can easily be dismissed as not being appropriate or representative for the specific situation. The same applies to estimates for the success probability and return rates. Other variables that significantly influence the resulting price, such as the number of patients treated, are challenging to predict accurately at the time a treatment is introduced to the market. A provisional comparison between the predicted patient numbers prior to market access and the patient numbers after the reimbursement decision, as reported by ZIN, suggests that actual patient figures can deviate significantly from initial projections.

A more implicit assumption that underlies these models that may still stand in the way of their actual implementation is that of a predetermined and unchanging market landscape. The aim of these models is to predetermine a price that allows known costs to be recouped with a fixed profit. However, scientific or market developments can readily undermine the entire underlying premise of certainty for payer and MAH that is the appeal of these models. This is demonstrated by the fact that three out of the thirteen therapies included in this study are registered for the treatment of spinal muscular atrophy in a span of less than five years, which is considerably shorter than the time horizons that the CPP and DCF model operate on.

### Strengths and weaknesses

This paper aimed to identify challenges in CEA and value-based pricing for orphan drugs and to test two recently proposed alternative pricing approaches. The strength of this study lies in the verification of problems and application of solutions to orphan drug pricing found in literature, rather than assessment of the quality of the CEA’s themselves.

Certain limitations of this study need to be addressed. The scope of this paper was limited to non-oncological orphan drugs that have received a formal assessment of cost-effectiveness by ZIN. This also means that these assessments adhere to the quality standards, criteria and perspective of ZIN which may vary from other HTA agencies. Moreover, the strong assumptions that underlie these pricing models also apply to the price estimates performed in this study. Although these are inherent to the pricing approaches, they significantly complicate the interpretability and reliability of the prices and highlighting the need for cautious application and thorough contextualization.

### Future research

Although the methodological issues highlighted in this research could potentially be addressed through additional data collection, either prior to or after reimbursement, or through the development of more refined analytical tools, it is questionable whether an HTA agency in a small country such as the Netherlands could realistically impose such requirements on MAHs. More importantly, these technical solutions would not resolve the second, more fundamental, concern: that these therapies have a very low probability of being cost-effective. The authors therefore concur with the recommendations from a recent Dutch review of pricing models, including value-based pricing, CPP and DCF, which emphasizes the need for future research to improve transparency around the true cost and risks associated with the development of new (orphan) drugs and creation of integrated differential pricing frameworks [31]. Such insights could be valuable to the pharmaceutical industry, payers, and decision-makers as they navigate the complex and often contentious process of ensuring patient access to necessary treatments.

## Conclusion

Challenges in CEA cited in literature are present in Dutch HTA reports for orphan drugs. Although these issues cause considerable uncertainty, they did not negate CEA’s current ability to inform decision-making. Still, orphan drugs tend to be far from cost-effective, providing a challenge for patient access that is both timely and financially feasible. It is unlikely that current alternative pricing models in the form of CPP and DCF will provide a solution to this issue as they tend to estimate lower prices for these drugs than CEA and involve significant assumptions and uncertainty.

## Electronic supplementary material

Below is the link to the electronic supplementary material.

Supplementary Material 1

## Data Availability

The datasets used and/or analysed during the current study are available from the corresponding author on reasonable request.
