# Supplementary material for: Methodological challenges in Dutch HTA of non-oncological orphan drugs: a retrospective analysis and price comparison using different pricing models
Source: Orphanet J Rare Dis. 2026 Jan 8;21:39. doi: 10.1186/s13023-025-04181-6 (PMC12870408; doi:10.1186/s13023-025-04181-6)
Supplement: Supplementary file 1 — Supplementary Material 1 [file 13023_2025_4181_MOESM1_ESM.docx]

#### Appendix A. Input values per treatment

| **Treatment name** | **Patients treated over 10 year period** | **Patients treated reference** | **Patients treated comment** | **Months of treatment over 10 year period** | **Treatment cost** | **Treatment cost reference** | **Treatment cost comment** | **Innovation bonus** |
| --- | --- | --- | --- | --- | --- | --- | --- | --- |
| Avacopan | 10620 | ZIN report, page 74 | Year 3 estimate of 1062 yearly patients multiplied by 10, single year of treatment | 12 | € 250 p/m | AIM fair price model | Orphan chemical | 15% |
| Atidarsagene autotemcel | 20 | ZIN report, page 227 | Estimated 2 patients per year multiplied by 10 | 1 | € 100.000 | AIM fair price model | Gene therapy | 40% |
| Pegcetacoplan | 24 | ZIN report, page 80-82 | Year 3 estimate, chronic use | 120 | € 750 p/m | AIM fair price model | Orphan biological | 25% |
| Risdiplam | 302 | ZIN report, page 92-96 | Year 3 estimate for both < 25 and > 25 years old, chronic use | 120 | € 250 p/m | AIM fair price model | Orphan chemical | 30% |
| Cannabidiol | 1135 | ZIN report, page 89 | Year 3 estimate, chronic use | 120 | € 250 p/m | AIM fair price model | Orphan chemical | 15% |
| Tafamidis | 2694 | ZIN report, page 71-72 | Average of year 3 estimates of scenario 1 and scenario 2, chronic use | 120 | € 250 p/m | AIM fair price model | Orphan chemical | 10% |
| Onasemnogene abeparvovec | 100 | ZIN report, page 236 | Year 3 estimate of 10,24 yearly patients multiplied by 10 | 1 | € 100.000 | AIM fair price model | Gene therapy | 40% |
| Ivacaftor/ tezacaftor /elexacaftor | 806 | ZIN report, page 78-80 | Year 3 estimate, chronic use | 120 | € 250 p/m | AIM fair price model | Orphan chemical | 20% |
| Givosiran | 18 | ZIN report, page 31-32 | Average of estimated 15-21 per year, chronic use | 120 | € 750 p/m | AIM fair price model | Orphan biological | 25% |
| Tezacaftor / ivacaftor | 131 | ZIN report, page 65-67 | Year 3 estimate, chronic use | 120 | € 250 p/m | AIM fair price model | Orphan chemical | 10% |
| Nusinersen | 200 | ZIN report, page 175-195 | Average of year 3 estimates of scenario 1 and scenario 3, chronic use | 120 | € 750 p/m | AIM fair price model | Orphan biological | 35% |
| Lumacaftor / ivacaftor | 498 | ZIN report, page 76-77 | Year 3 estimate, chronic use | 120 | € 250 p/m | AIM fair price model | Orphan chemical | 10% |

#### Appendix B. Results in Table 3 as percentages of the value-based price (input values for Figure 2)

|  | **CPP price** | | **DCF price** | | **Value-based price** | **List price** |
| --- | --- | --- | --- | --- | --- | --- |
| **Substance name** | **Min** | **Max** | **Min** | **Max** | **ZIN** | **MAH** |
| Avacopan | 31% | 78% | 34% | 121% | 100% | 500% |
| Atidarsagene autotemcel 1 | 142% | 1107% | 235% | 2059% | 100% | 667% |
| Atidarsagene autotemcel 2 | 53% | 415% | 88% | 772% | 100% | 250% |
| Pegcetacoplan | 98% | 756% | 161% | 1396% | 100% | 667% |
| Risdiplam 1 | 45% | 212% | 60% | 374% | 100% | 1667% |
| Risdiplam 2 | 12% | 58% | 16% | 102% | 100% | 455% |
| Cannabidiol | 18% | 44% | 20% | 67% | 100% | 125% |
| Tafamidis | 4% | 6% | 4% | 9% | 100% | 125% |
| Onasemnogene abeparvovec | 137% | 613% | 180% | 1079% | 100% | 1111% |
| Ivacaftor/ tezacaftor /elexacaftor 1 | 9% | 25% | 10% | 40% | 100% | 400% |
| Ivacaftor/ tezacaftor /elexacaftor 2 | 7% | 21% | 8% | 34% | 100% | 333% |
| Givosiran | 18% | 143% | 30% | 265% | 100% | 167% |
| Tezacaftor / ivacaftor | 25% | 162% | 37% | 292% | 100% | 500% |
| Voretigene neparvovec | n.a. | n.a. | n.a. | n.a. | n.a. | n.a. |
| Nusinersen 1 | n.a. | n.a. | n.a. | n.a. | n.a. | n.a. |
| Nusinersen 2 | 5% | 15% | 5% | 26% | 100% | 67% |
| Lumacaftor / ivacaftor | 16% | 60% | 20% | 101% | 100% | 556% |
